# Platelet microRNAs as Potential Novel Biomarkers for Antiplatelet Therapy with P2Y_12_ Inhibitors and Their Association with Platelet Function

**DOI:** 10.3390/jcm13010063

**Published:** 2023-12-22

**Authors:** Karolina Gumiężna, Adrian Bednarek, Grażyna Sygitowicz, Agata Maciejak-Jastrzębska, Piotr Baruś, Jaromir Hunia, Dominika Klimczak-Tomaniak, Janusz Kochman, Marcin Grabowski, Mariusz Tomaniak

**Affiliations:** 1 First Department of Cardiology, Medical University of Warsaw, Banacha 1a, 02-097 Warsaw, Poland; kgumiezna@gmail.com (K.G.);; 2 Department of Clinical Chemistry and Laboratory Diagnostics, Medical University of Warsaw, 02-097 Warsaw, Poland; gsygitowicz@poczta.onet.pl (G.S.); agata.maciejak@wum.edu.pl (A.M.-J.); 3 Department of Cardiology, Hypertension and Internal Medicine, Medical University of Warsaw, 02-097 Warsaw, Poland; 4 Department of Immunology, Transplantation and Internal Medicine, Medical University of Warsaw, 02-097 Warsaw, Poland

**Keywords:** acute coronary syndrome, dual antiplatelet therapy, microRNA, platelet reactivity, P2Y_12_ inhibitors

## Abstract

Introduction: Patients with acute coronary syndrome (ACS) undergoing percutaneous coronary intervention (PCI) require dual antiplatelet therapy (DAPT). However, the response to treatment can vary considerably. Certain platelet microRNAs (miRs) are suspected to predict DAPT response and influence platelet function. This study aimed to analyze selected miRs’ expressions and compare them among patients treated with different P2Y_12_ inhibitors while assessing their association with platelet activity and turnover parameters. Materials and methods: We recruited 79 ACS patients post-PCI treated with clopidogrel, ticagrelor, or prasugrel, along with 18 healthy volunteers. Expression levels of miR-126-3p, miR223-3p, miR-21-5p, miR-197-3p, and miR-24-3p, as well as immature platelet fraction (IPF) and ADP-induced platelet reactivity, were measured and compared between groups. Results: Analyses revealed significantly lower expressions of miR-126-3p, miR-223-3p, miR-21-5p, and miR-197-3p in patients treated with ticagrelor, compared to clopidogrel (fold changes from −1.43 to −1.27, *p*-values from 0.028 to 0.048). Positive correlations were observed between platelet function and the expressions of miR-223-3p (r = 0.400, *p* = 0.019) and miR-21-5p (r = 0.423, *p* = 0.013) in patients treated with potent drugs. Additionally, miR-24-3p (r = 0.411, *p* = 0.012) and miR-197-3p (r = 0.333, *p* = 0.044) showed correlations with IPF. Conclusions: The identified platelet miRs hold potential as biomarkers for antiplatelet therapy. (ClinicalTrials.gov number, NCT06177587).

## 1. Introduction

Dual antiplatelet therapy (DAPT) is a standard treatment for patients with acute coronary syndrome (ACS) who undergo percutaneous coronary intervention (PCI). It consists of acetylsalicylic acid (ASA) and the inhibitor of the P2Y_12_ receptor, which most commonly is either clopidogrel, ticagrelor, or prasugrel. This treatment is crucial for the prevention of stent thrombosis (ST) or recurrent myocardial infarction (MI), as well as for the reduction in the long-term risk of cardiovascular (CV) death [1].

However, even 30% of the population can show inadequate platelet inhibition, resulting in high on-treatment platelet reactivity (HTPR) during DAPT with clopidogrel, which is associated with a greater risk of ST or CV events [2,3,4]. The variability in responses to prasugrel and ticagrelor is lower than in responses to clopidogrel but still present [5,6]. However, little is known about the indicators that can predict this state.

Unfortunately, the tests measuring platelet reactivity available so far are not routinely performed before or during DAPT, and they appear in ESC guidelines with a low class of recommendation (class IIb), due to multiple limitations [7,8]. Therefore, there is still a need for novel biomarkers that would be useful in predicting the risk of HTPR occurrence, thus enabling better individual DAPT strategy planning and monitoring.

MicroRNAs (miRs) are short (18–25 nucleotides), non-coding RNAs that regulate gene expression. Many of them target messenger RNAs (mRNAs) that encode proteins involved in platelets’ activation and aggregation, thereby influencing the regulation of their expression. Some studies suggest that the level of the most abundant platelet-expressed miRs may be a potential marker of antiplatelet reactivity and response, yet their importance is still unclear and requires further research [9].

The primary aim of our study was to evaluate the correlation between DAPT with the use of different P2Y_12_ inhibitors and the expressions of selected miRs: miR-126-3p, miR-21-5p, miR-223-3p, miR-197-3p, and miR-24-3p. Moreover, we attempted to assess their role in the regulation of platelet function and turnover by investigating their relationship with ADP-induced platelet aggregometry and immature platelet fraction (IPF) levels.

## 2. Materials and Methods

### 2.1. Population

This was a prospective, single-center study performed in the 1st Chair and Department of Cardiology, Medical University of Warsaw. Seventy-nine patients who underwent PCI and received DAPT were recruited between March 2016 and May 2018. DAPT consisted of a low dose (75 mg) of ASA in all patients and either 75 mg clopidogrel once a day (n = 40), 90 mg ticagrelor twice a day (n = 21), or 10 mg of prasugrel daily (n = 18). Before PCI, patients received 300 mg of ASA and the loading dose of the P2Y_12_ inhibitor (either 300 mg of clopidogrel, 180 mg of ticagrelor, or 60 mg of prasugrel). The inclusion criteria were: age > 18 years old, diagnosis of ACS, and treatment with PCI during hospitalization, followed by DAPT. The patients were excluded if informed consent could not be obtained or if the miR expression was not assessed. Moreover, the exclusion criteria were: other treatment that had an impact on platelet function or coagulation, including ongoing DAPT prior to admission; low (<30%) or high (>52%) hematocrit; and low platelet count (<100 × 10^9^/L). The control group (n = 18) was chosen from among healthy individuals with no history of coronary artery disease and no documented intake of medications affecting platelet function or coagulation currently or in the past. Blood samples for all the measurements were collected in the first 24 h after the PCI. Blood was collected in the catheterization laboratory before patients were moved to the ward, with more than 90% of cases involving collection after approximately two hours after the PCI procedure. The study was conducted according to the principles outlined in the Declaration of Helsinki. Approval from the local medical ethics committee was obtained. The study was registered with ClinicalTrials.gov under the identifier NCT06177587.

### 2.2. Sample Processing and miRNA Isolation

Whole blood samples were collected from each individual into serum separator tubes using standard phlebotomy techniques. Serum was fractionated from whole blood samples following the manufacturer’s protocol and was stored at −80 °C prior to the analysis. The serum samples were analyzed using spectrophotometry to be free from hemoglobin [10].

Total RNA was isolated from 200 μL of serum using miRCURYTM RNA Isolation Kit–Biofluids (Exiqon A/S, Vedbaek, Denmark), with DNAse digestion on-column. For increased reproducibility and for quality control of extraction efficiency, carrier RNA from the bacteriophage MS2 (Roche Diagnostics GmbH, Mannheim, Germany) and a mix of three synthetic control templates (RNA Spike-in mix UniSp2, UniSp4, and UniSp5; Exiqon A/S) were added to the samples just before purification.

### 2.3. Quantitative Real-Time RT-PCR and Data Analyzes

First-strand cDNA synthesis was performed using the miRCURY LNA™ Universal RT microRNA PCR, Universal cDNA Synthesis Kit II (Exiqon A/S), with the addition of a mixture of two synthetic spike-ins, UniSp6 and cel-miR-39-3p (Exiqon A/S). The expression levels of selected miRNAs (has-miR-126-3p, has-miR-21-5p, has-miR-223-3p, has-miR-197-3p, has-miR-24-3p, and miR-21-5p) and potential reference candidates (has-let-7i-5p and has-miR-103a-3p) were evaluated using microRNA LNATM PCR primer sets and ExiLENT SYBR^®^ Green Master Mix (Exiqon A/S on the LightCycler^®^ 480 Real-Time PCR system (Roche Diagnostics, Basel, Switzerland)). The amplification curves were analyzed using the Roche LC software (version 1.5), both for the determination of the crossing-point (Cp) values (by the second derivative method) and for melting curve analysis. RT-qPCR data were normalized to let-7i-5p, which was the most stable reference miR. The expression level of each miR was represented by the fold change, which was calculated using the REST 2009 software (Relative Expression Software Tool V2.0.13, Qiagen GmbH (Hilden, Germany)) [11].

### 2.4. Platelet Reactivity and Other Blood Parameters

Blood samples were drawn from the peripheral vein and collected into hirudin-containing tubes. Impedance aggregometry by Multiplate^®^ Analyzer (Roche Diagnostics, Basel, Switzerland) using adenosine diphosphate (ADP) as the agonist was performed 30–120 min after sampling. Maximum platelet aggregation and aggregation velocity were expressed in arbitrary units (AUC, aggregation units [AU] × minutes). Platelet count, hemoglobin, white blood cell count, platelet distribution width, mean platelet volume (MPV), and IPF were assessed in whole blood anticoagulated with ethylenediaminetetraacetic (K3EDTA) using an automated hematological analyzer (Sysmex XN 2000, Kope, Japan).

### 2.5. Statistical Methods

Statistical analysis was performed using SPSS Statistics 28.0 (IBM, New York, NY, USA). For continuous variables, the Shapiro–Wilk test was carried out to assess the normality of distribution. The parametric variables were presented as means and standard deviations (SD), while parameters with distributions other than normal were presented as the median along with the interquartile range (IQR), encompassing the 25th and 75th percentiles. The differences between groups were compared using ANOVA or the Kruskal–Wallis test as appropriate. Categorical variables were presented as numbers with percentages and compared using the Chi-square test. Correlation analyses between non-parametric variables were performed with Spearman’s rho. All reported *p*-values were two-sided, and *p* < 0.05 was considered significant.

## 3. Results

### 3.1. Population

A total of 97 patients were enrolled in the study, including 79 patients after PCI receiving DAPT, and 18 healthy volunteers. The mean age in the study group was 61.3 years. A total of 61 patients (77.2%) were male, 21 (26.6%) were diagnosed with diabetes mellitus, 60 (75.9%) had hyperlipidemia, 43 (54.5%) had a history of smoking (including past and current smokers), and 17 (21.5%) had a history of previous MI. The study group was divided based on the P2Y_12_ inhibitor they received. The mean ages were 66.1, 60.3, and 56.1 years in the clopidogrel, ticagrelor, and prasugrel groups, respectively. Apart from that, there were no significant differences between the groups. The baseline characteristics are presented in Table 1.

### 3.2. Comparison of miR Expression between Groups

**Figure 1:**
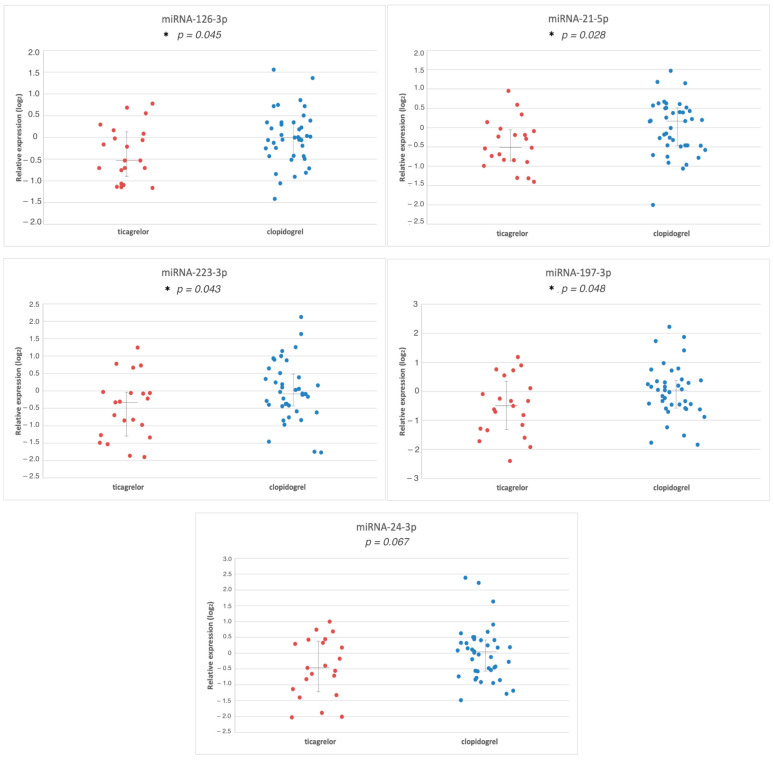
The relative expressions of microRNAs: log2 ratio of fold change (normalized to let-7i-5p). *p*-value < 0.05 was considered statistically significant (*). The lines represent the median fold change in the 25th and 75th percentiles.

**Figure 2:**
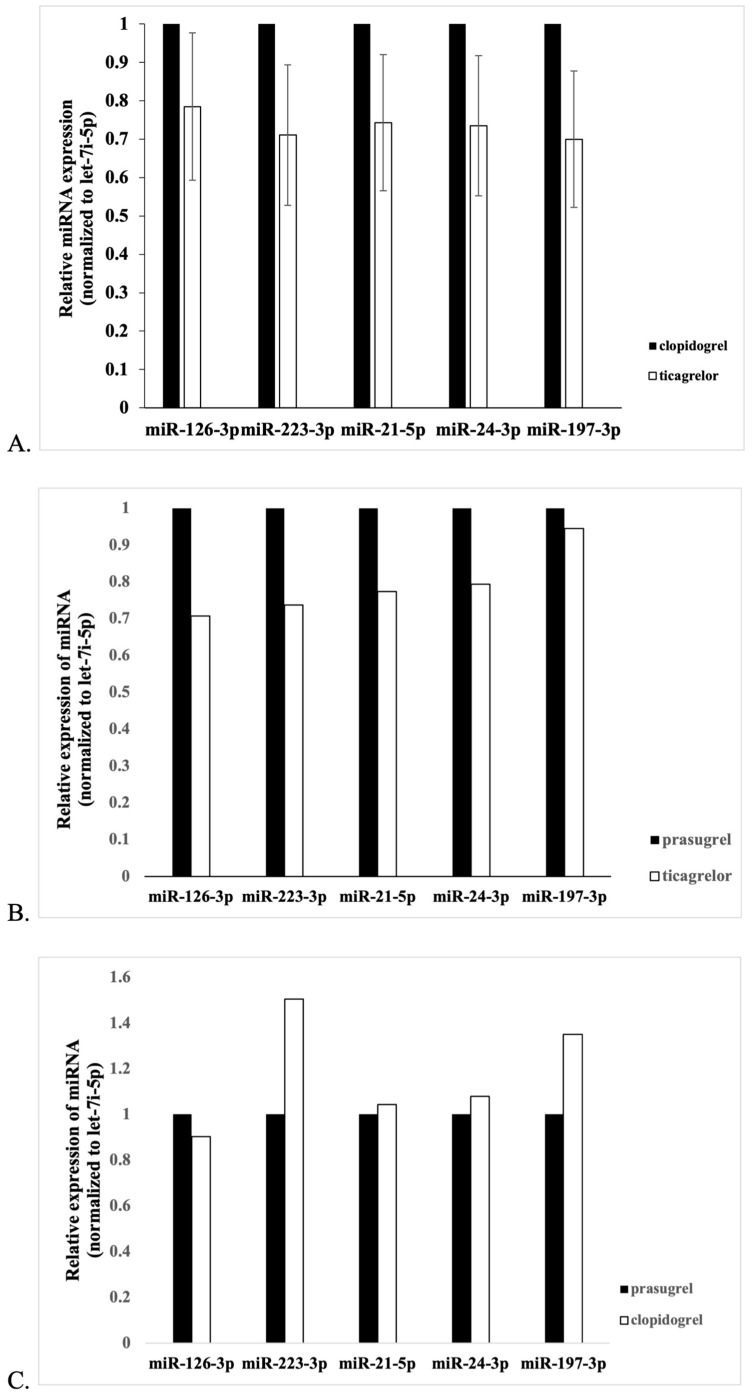
Relative expressions of miRNAs between investigated groups. Results were determined by RT-qPCR and normalized to let-7i-5p. Data are presented as expression ratio ± standard error. (**A**) Significant differences (*p*-value < 0.05) for miR-126-3p, miR-223-3p, miR-21-5p, and miR-197-3p. (**B**) Significant differences (*p*-value < 0.05) for miR-126-3p. (**C**) Significant differences (*p*-value < 0.05) for miR-223-3p

Among five investigated miRs, the levels of four were statistically significantly decreased in ticagrelor- vs. clopidogrel-treated patients (Figure 1 and Figure 2). They included miR-126-3p (fold change −1.27, *p* = 0.045), miR223-3p (fold change −1.41, *p* = 0.043), miR-21-5p (fold change −1.35, *p* = 0.028), and miR197-3p (fold change −1.43, *p* = 0.048). The level of miR24-3p was also reduced; however, the difference did not reach statistical significance (fold change −1.36, *p* = 0.067). Moreover, patients treated with ticagrelor manifested a decreased expression of miR-126-3p (fold change −1.41, *p* = 0.031), and patients treated with clopidogrel manifested an increased expression of miR-223-3p (fold change 1.51, *p* = 0.023) when compared to the prasugrel-treated group (Figure 2). There were no other statistical differences in miR expression for the prasugrel-treated group; however, due to the small size of this population, they cannot be definitely excluded.

Only the serum levels of miR-126-3p and miR-21-5p were statistically significantly altered in patients treated with clopidogrel and ticagrelor, compared with the control group. The expression level of miR-126-3p was significantly decreased in patients treated with clopidogrel versus the control group (fold change −1.37, *p* = 0.010) and in patients treated with ticagrelor versus the control group (fold change −1.75, *p* < 0.001). The expression level of miR-21-5p was down-regulated in patients treated with ticagrelor versus the control group (fold change −1.65, *p* = 0.003).

### 3.3. Expression of miR vs. Platelet Function and Platelet Turnover

The impedance aggregometry with ADP was performed in 63 patients treated with DAPT. Among them, 29 were treated with clopidogrel and 34 with a potent P2Y_12_ inhibitor (19 with ticagrelor and 15 with prasugrel). The latter was pooled into one group, due to the lack of significant differences in ADP-induced platelet aggregometry levels in previous studies between ticagrelor- and prasugrel-treated patients [12]. We observed a positive correlation between platelet function and the expressions of the following particles: miR-223-3p (r = 0.400 CI: 0.062–0.656, *p* = 0.019) and miR-21-5p (r = 0.423 CI: 0.088–0.671, *p* = 0.013) in patients treated with a potent medication. The IPF level was measured in 37 patients (treated with clopidogrel or ticagrelor), and a correlation was found only with miR-24-3p (r = 0.411 CI: 0.090–0.654, *p* = 0.012) and miR-197-3p (r = 0.333 CI: 0.0–0.6, *p* = 0.044). Complete results of the correlation analyses are shown in Table 2.

Based on ADP levels, all the patients were divided into three subgroups: hyperresponders (AUC < 21), normoresponders (AUC 21–47), and hyporesponders (AUC > 47). The analysis of the expression of studied miRs between groups revealed that the levels of miR-126-3p and miR-21-3p differed significantly among groups. The expression of miR-126 was higher in normoresponders than in both hyper- (*p* = 0.021) and hyporesponsive patients (*p* = 0.040). MiR-21-5p was also the highest in the middle group (*p* = 0.005 and *p* = 0.027 for greater and inadequate response, respectively). Other differences inside the groups included lower expressions of miR-223 and miR-24 in hyperresponders than in normoresponders, with no significant differences when compared with the HTPR group.

## 4. Discussion

The presented study assessed the expression of selected miRs involved in platelet activation depending on selected pharmacological treatment in ACS patients. MiR-126, miR-21, miR-223, miR-197, and miR-24 are the most highly expressed miRs in platelets and can regulate protein production, affecting platelet activation [13]. The results of our study could be summarized as follows: (i) DAPT is associated with the decreased levels of several platelet miRs; (ii) the choice of P2Y_12_ inhibitor possibly has an impact on miR levels; (iii) expressions of miR-223-3p and miR-21-5p showed correlation with platelet function in patients treated with potent P2Y_12_ inhibitors (the higher the expression of miRs, the higher the ADP-induced platelet aggregation level) but not with clopidogrel; and (iv) miR-24-3p and miR-197-3p showed correlation with the platelet turnover marker (i.e., IPF).

MiR-223 is one of the most abundantly expressed platelet miRs responsible for down-regulating the expression of the P2Y_12_ receptor gene (*P2RY12*). It was shown to target 3′UTR of P2Y_12_ mRNA [14]. Opposite to some previous works, we described its lower expression in high responders [15,16]. However, previous works differed from ours in several aspects, including the choice of study group, the selection of the P2Y_12_-inhibiting agent, and the test used for platelet reactivity assessment. The existing research examining the relationship between miR-223 and platelet reactivity during treatment has predominantly concentrated on the use of clopidogrel therapy. We showed that the relationship was present in patients treated with other P2Y_12_ inhibitors, though in an opposite direction. Spearman correlation analysis revealed that in a group of patients treated with a potent inhibitor, that is, ticagrelor or prasugrel, lower miR-223 expression was associated with decreased platelet reactivity. Also, the use of a potent drug was associated with its lower expression than in clopidogrel-treated individuals. In our patient cohort, measurements were conducted on the first day, usually after 2 h following the administration of a saturating dose. We suspect that the more effective inhibition of the P2Y_12_ receptor during this initial period could be primarily attributed to the downregulation of miR-223 expression, serving as a potential feedback mechanism to regulate platelet activity.

Interestingly, miR-21 function has been defined both as protective and impairing on endothelial function, depending on the conditions [17,18]. The exact pathways that are involved in this process have not been clearly defined yet, due to the multiplicity of the target genes, but previous reports revealed an association of its level with recurrent venous thromboembolism [19]. The correlation analysis also revealed that a higher expression of miR-21 is associated with greater platelet reactivity in patients treated with a potent inhibitor. Its functional role in platelets and megakaryocytes was shown before [20], indicating that its levels correlate with several platelet-derived profibrotic factors and may be related to platelet count. That stays in line with our finding showing a lower expression of miR-21 in hyperresponsive patients, as high responsiveness may be related both to the decreased production of profibrotic particles or to reduced platelet formation. And conversely, higher levels of miR-21 could cause greater platelet reactivity. However, the expression of this particle was also the highest in the normal-response group, a finding that remains without known justification at this time.

Platelet activation and aggregation play crucial roles in the processes underlying cardiovascular disorders. Insufficient platelet inhibition when on DAPT may result in higher rates of adverse events, such as MI and CV death. Previous analyses revealed that the expression patterns of three specifically chosen miRs served as effective indicators for predicting MI. Notably, miR-126 exhibited a positive association with the incidence of MI, whereas miR-223 and miR-197 displayed an inverse relationship in this context [21]. As we showed, miR-223 expression was the highest in normoresponders, namely patients with the most well-balanced treatment platelet reactivity. That might explain the higher rates of adverse cardiovascular events in groups with lower levels of miR-223. However, we observed the same trend considering miR-126, though the views of previous studies remain unclear. On the other hand, Hromadka et al. observed opposite findings, i.e., the higher the miR-223 level, the greater the risk of acute MI. Additionally, the investigation identified that individuals at the utmost risk of experiencing MI were those characterized by elevated expression levels of miR-223 and concurrently diminished levels of miR-126, as assessed by their respective ratios [22]. In the view of presented findings, the roles of miR-223 and miR-126 in predicting MACE are evident but not fully understood. It should also be remembered that other phenomena, such as platelet exhaustion, may have an impact on the presented results; however, a dedicated study focused on a particular mechanism ought to be performed.

The expression of miRs was also dependent on the chosen treatment. Opposite to a previous preliminary study [23], we showed that the expression of miR-223 differed significantly among treatment groups, being higher in clopidogrel than both ticagrelor- and prasugrel-treated patients. However, this preliminary study was performed on a smaller cohort of 21 patients (10 and 11 patients in subgroups) and, contrary to our study, excluded patients with STEMI. Based on the outcomes of the correlation analysis, which demonstrated that the relationship existed for ticagrelor- or prasugrel-treated patients only, we assumed that potent P2Y_12_ inhibitors may have a greater impact on miR-223 expression than clopidogrel. However, the comparison between treated patients and the control group revealed that miR-126-3p expression is influenced by clopidogrel treatment as well. Contrasting these results with the study by Willeit et al. allows us to find similarity in terms of the effect of clopidogrel therapy on miR-126 expression and the lack of effect on miR-21, miR-24, and miR-197 [13]. The only difference remains in terms of miR-223, which was influenced by clopidogrel treatment in a previous study but not in our study. Willeit et al. also found that with stronger inhibition, the level of miRs is more affected by the therapy. The findings presented above allow us to partially confirm that the use of ticagrelor caused a more significant reduction in miR levels than clopidogrel treatment. Unfortunately, we performed measurements at one time point (at the start of treatment within 24 h after PCI, usually about 2 h after the initial loading dose), which did not allow us to verify the impact of the duration of therapy on changes in miRs expressions. Moreover, we showed that there was not only a significant difference between clopidogrel and new P2Y_12_ inhibitors, but also that the level of miR-126 was reduced in ticagrelor when compared to prasugrel-treated patients. The groups treated with each drug did not differ significantly in characteristics apart from age. Simon et al. conducted an investigation into the relationship between age and the most highly expressed platelet miRs among other factors. Their findings did not reveal any evidence suggesting differential expression of the studied particles based on age [24]. Therefore, we suspect that this difference did not significantly affect the results we obtained.

Antiplatelet therapy plays a crucial role in the prevention of ST or recurrent MI in patients after PCI. However, the response to antiplatelet drugs varies among patients; thus, an individualized approach in pharmacologic management is warranted, with it being a cornerstone for maintaining a perfect balance between bleeding and thrombosis risk. Evidence suggests that platelet miRs are stable in serum and plasma, which translates into a good correlation of the level of circulating miRs with their platelet expression. Considering our current discoveries and the findings from previous research, it is reasonable to entertain the notion that platelet miRs could serve not only as reliable predictors of the response to antiplatelet therapy but also as indicative markers for precise monitoring. Nevertheless, to thoroughly assess their correlation with cardiovascular outcomes, further studies involving the measurement of miR levels during therapy are warranted.

While our study provides valuable insights, it is essential to acknowledge certain limitations inherent in our research. A notable constraint was the relatively small size of the patient group under investigation. Moreover, the measurements of platelet reactivity were not obtained for all the patients, which carries the risk of bias. However, the doctors and nurses included in the diagnostic and therapeutic processes as well as the researchers obtaining blood for platelet reactivity measurements were blinded to the results of the miR expression in order to minimize this risk. Finally, the measurements of all the studied parameters were performed only at one time point, which did not allow us to compare miR association with the response to treatment over time. We, however, find it interesting in the light of our findings and believe that future research should also focus on this aspect.

## 5. Conclusions

The presented study showed how the most highly expressed platelet miRs are affected by the application of DAPT and the choice of P2Y_12_ inhibitor. Moreover, the expressions of several miRs showed a relationship with platelet function. If confirmed on a larger cohort, the findings may contribute to figuring out a way to use platelet miRs as biomarkers for guiding antiplatelet therapy.

## Figures and Tables

**Table 1:** Baseline characteristics. Bold values represent statistical significance at the *p* < 0.05 level. Abbreviations: ACEI—angiotensin-converting enzyme inhibitor; ARB—angiotensin II receptor blocker; BB—beta blocker; CABG—coronary artery bypass grafting; CCB—calcium channel blocker; DM—diabetes mellitus; HL—hyperlipidemia; Hgb—hemoglobin; HT—hypertension; LVEF—left ventricle ejection fraction; MI—myocardial infarction; n—number; PCI—percutaneous coronary intervention; PLT—platelet count; PPI—proton pump inhibitor; SD—standard deviation; STEMI—ST-elevation myocardial infarction; y—years.

	All (n = 79)	Clopidogrel (n = 40)	Ticagrelor (n = 21)	Prasugrel (n = 18)	*p*-Value
Mean age, y (SD)	61.3 (11.7)	66.1 (9.2)	60.3 (12.2)	56.1 (12.4)	**0.002**
Male sex, n (%)	61 (77.2)	30 (75.0)	15 (71.4)	16 (88.9)	0.386
HT, n (%)	58 (73.4)	29 (72.5)	14 (66.7)	15 (83.3)	0.493
DM, n (%)	21 (26.6)	11 (27.5)	3 (14.3)	7 (38.9)	0.219
HL, n (%)	60 (75.9)	34 (85.0)	16 (76.2)	10 (55.5)	0.053
Past smokers, n (%)	16 (20.3)	11 (27.5)	2 (9.5)	3 (16.7)	0.230
Current smokers, n (%)	27 (34.2)	10 (25.0)	10 (47.6)	7 (38.9)	0.186
Previous MI, n (%)	17 (21.5)	9 (22.5)	5 (23.8)	3 (16.7)	0.844
Previous PCI, n (%)	19 (24.1)	11 (27.5)	6 (28.6)	2 (11.1)	0.342
Previous CABG, n (%)	3 (3.8)	1 (2.5)	2 (9.5)	0 (0.0)	0.249
Mean LVEF, % (SD)	49.1 (10.2)	49.0 (11.7)	47.9 (7.9)	50.3 (9.4)	0.818
STEMI	44 (55.7)	22 (55.0)	10 (47.6)	12 (66.7)	0.487
Statin, n (%)	79 (100)	40 (100)	21 (100)	18 (100)	1.0
BB, n (%)	69 (87.3)	37 (92.5)	18 (85.7)	14 (77.8)	0.286
ACEI/ARB, n (%)	74 (93.7)	38 (95.0)	20 (95.2)	16 (88.9)	0.637
CCB, n (%)	12 (15.2)	8 (20.0)	2 (9.5)	2 (11.1)	0.479
PPI, n (%)	74 (93.7)	37 (92.5)	20 (95.2)	17 (94.4)	0.906
Creatinine [mg/dL] median (25; 75 percentile)	0.94 (0.82; 1.13)	0.97 (0.84; 1.15)	0.91 (0.81; 1.12)	0.94 (0.80; 1.1)	0.831
Hgb [g/dL] median (25; 75 percentile)	13.8 (12.4; 14.6)	13.6 (12.2; 14.7)	13.9 (12.2; 14.4)	14.3 (12.4; 15.5)	0.662
PLT 10^3^/μL median (25; 75 percentile)	220 (194; 252)	215 (201; 250)	223 (192; 265)	227 (176; 271)	0.336

**Table 2:** The results of correlation analyses performed for selected micro-RNAs and platelet functions measured with impedance aggregometry, with ADP used as the agonist and with platelet turnover represented by IPF. Bold values represent statistical significance at the *p* < 0.05 level. Abbreviations: IPF—immature platelet fraction, miR—micro-RNA, rho—Spearman’s rho, *—*p* < 0.05.

	miR-126-3p		miR-223-3p		miR-21-5p		miR-24-3p		miR-197-3p	
	*p*-Value	Rho	*p*-Value	Rho	*p*-Value	Rho	*p*-Value	Rho	*p*-Value	Rho
Aggregometry (n = 63)	0.600	0.067	0.119	0.199	0.128	0.194	0.363	0.117	0.324	0.126
Aggregometry, clopidogrel (n = 29)	0.468	−0.14	0.971	−0.007	0.491	−0.133	0.909	−0.022	0.984	−0.004
Aggregometry, potent P2Y_12_ inhibitors (n = 34)	0.271	0.194	0.019 *	**0.400**	0.013 *	**0.423**	0.196	0.227	0.212	0.220
Aggregometry, ticagrelor (n = 19)	0.661	0.125	0.180	0.321	0.068	0.427	0.811	0.059	0.668	0.105
Aggregometry, prasugrel (n = 14)	0.850	0.054	0.930	−0.025	0.732	0.096	0.685	0.114	0.732	−0.096
IPF (n = 37)	0.295	0.177	0.256	0.126	0.391	0.145	0.012 *	**0.411**	0.044 *	**0.333**

## Data Availability

The data presented in this study are available on request from the corresponding author.
